# Supplementary material for: Time trends, factors associated with, and reasons for COVID-19 vaccine hesitancy: A massive online survey of US adults from January-May 2021
Source: PLoS One. 2021 Dec 21;16(12):e0260731. doi: 10.1371/journal.pone.0260731 (PMC8691631; doi:10.1371/journal.pone.0260731)
Supplement: S1 Appendix — (PDF) [file pone.0260731.s001.pdf]

sAppendix. Carnegie Mellon University (CMU) Delphi Group's COVID Trends and Impact Survey (CTIS): select questions and response sets.

Below are the survey items used in this study. Unless otherwise noted, the survey items remained unchanged during the study period. The full text of each survey instrument is available online: https://cmu-delphi.github.io/delphi-epidata/symptom-survey/coding.html

D1 What is your gender?

1. Male
2. Female
3. Non-binary
4. Prefer to self-describe ________________
5. Prefer not to answer

D2 What is your age?

1. 18-24 years
2. 25-34 years
3. 35-44 years
4. 45-54 years
5. 55-64 years
6. 65-74 years
7. 75 years or older

D6 Are you of Hispanic, Latino, or Spanish origin?

1. Yes
2. No, not of Hispanic, Latino, or Spanish origin

D7 What is your race? Please select all that apply.

1. American Indian or Alaska Native
2. Asian
3. Black or African American
4. Native Hawaiian or other Pacific Islander
5. White
6. Some other race

D8 What is the highest degree or level of school you have completed?

1. Less than high school
2. High school graduate or equivalent (GED)
3. Some college
4. 2 year degree
5. 4 year degree
6. Master’s degree
7. Professional degree (e.g. MD, JD, DVM)
8. Doctorate

D9 In the past 4 weeks, did you do any kind of work for pay?

1. Yes
2. No

D10 [Displayed if D9 = “Yes”] Was any of your work for pay in the last four weeks outside your home?

1. Yes
2. No

A3 What is your current ZIP code? _____________

B11 Have you **ever** tested positive for coronavirus (COVID-19)?

1. Yes
2. No
3. I don't know

C1 Have you ever been told by a doctor, nurse, or other health professional that you have any of the following medical conditions? Please select all that apply.

1. Cancer (other than skin cancer)
2. Heart attack, heart disease, or other heart condition
3. High blood pressure
4. Asthma
5. Chronic lung disease such as COPD, chronic bronchitis, or emphysema
6. Kidney disease
7. Autoimmune disorder such as rheumatoid arthritis or Crohn’s disease
8. Type 1 diabetes
9. Type 2 diabetes
10. Weakened or compromised immune system
11. Obesity
12. None of these

A5 How many people, including you, are currently staying in your household?

1. Children under 18 years old _____________
2. Adults between 18 and 64 years old _____________
3. Adults 65 years old or older ______________

C9 How worried do you feel that you or someone in your immediate family might become seriously ill from COVID-19 (coronavirus disease)?

1. Very worried
2. Somewhat worried
3. Not too worried
4. Not worried at all

C17 Have you gotten a seasonal flu vaccine since June 2020?

1. Yes, I have gotten a seasonal flu vaccine since June 2020
2. No, I have not gotten a seasonal flu vaccine since June 2020
3. I don't know if I have gotten a seasonal flu vaccine since June 2020

C7 To what extent are you intentionally avoiding contact with other people?

1. All of the time
2. Most of the time; I only leave my home to buy food and other essentials
3. Some of the time; I have reduced the amount of times I am in public spaces, social gatherings, or at work
4. None of the time

V1 Have you had a COVID-19 vaccination?

1. Yes
2. No
3. I don't know

V3 If a vaccine to prevent COVID-19 were offered to you today, would you choose to get vaccinated?

1. Yes, definitely
2. Yes, probably
3. No, probably not
4. No, definitely not

V5

a) [Displayed if V3 = “Yes, probably”] Which of the following, if any, are reasons that you only probably will* get a COVID-19 vaccine? Please select all that apply.
b) [Displayed if V3 = “No, probably not”] Which of the following, if any, are reasons that you probably won’t* get a COVID-19 vaccine? Please select all that apply.
c) [Displayed if V3 = “No, definitely not”] Which of the following, if any, are reasons that you definitely won’t* get a COVID-19 vaccine? Please select all that apply.

*The wording of the response sets was changed from “will get” to “would choose to get,” and “won’t get” to “wouldn’t choose to get,” on February 8, 2021.

Answer choices:

1. I am concerned about possible side effects of a COVID-19 vaccine.
2. I am concerned about having an allergic reaction to a COVID-19 vaccine.
3. I don't know if a COVID-19 vaccine will work.
4. I don't believe I need a COVID-19 vaccine.
5. I don't like vaccines.
6. My doctor has not recommended it.
7. I plan to wait and see if it is safe and may get it later.
8. I think other people need it more than I do right now.
9. I am concerned about the cost of a COVID-19 vaccine.
10. I don't trust COVID-19 vaccines.
11. I don't trust the government.
12. It is against my religious beliefs.
13. I have a health condition and am concerned about the safety of the vaccine for people with my condition.
14. I am currently/planning to be pregnant and/or breastfeeding and do not want to get vaccinated at this time.
15. Other
