# Supplementary material for: Time trends, factors associated with, and reasons for COVID-19 vaccine hesitancy: A massive online survey of US adults from January-May 2021
Source: PLoS One. 2021 Dec 21;16(12):e0260731. doi: 10.1371/journal.pone.0260731 (PMC8691631; doi:10.1371/journal.pone.0260731)
Supplement: S1 Table — (PDF) [file pone.0260731.s002.pdf]

sTable 1. Participant flow for Carnegie Mellon University (CMU) Delphi Group's COVID Trends and Impact Survey (CTIS) by month (January-May, 2021)

	**January[a]**	**February**	**March**	**April**	**May [b]**	**Total**
	**N**					
Offered survey	106,387,320	95,953,902	104,768,154	103,399,752	66,138,989	476,648,117
Did not respond	105,073,194	94,721,211	103,476,197	102,315,236	65,576,417	471,162,255
Responded	1,314,126	1,232,691	1,291,957	1,084,516	562,572	5,485,862
Response rate	1.24%	1.28%	1.23%	1.05%	0.85%	1.15%
Did not report hesitancy	112,319	83,829	74468	61896	32,914	365,426
Reported self-describe gender[c]	6,157	6,667	7,953	6,873	4,014	31,664
**Report sample**	1,195,650	1,142,195	1,209,536	1,015,747	525,644	5,088,772
Vaccinated/Definitely Yes	x	x	x	x	435,393	x
Did not provide reasons	x	x	x	x	2,267	x
**Reasons sample**	x	x	x	x	87,984	x

[a] January data was subset to January 6-31 because January 6 was the first date both vaccine questions (receipt and intent) were both asked.

[b] May data was subset to include only those receiving the survey prior to an update to the vaccine questions; on May 20 a new survey version was offered to approximately 85% of potential respondents.

[c] A review of fill-in responses for self-described gender suggested the majority of participants who selected this category did not complete the survey in good faith (e.g., wrote-in discriminatory statements, and as a group had a high frequency of extreme responses such as the oldest age group and highest education level). Thus, they were excluded from the main analysis. They are included in a sensitivity analysis (sTable 8-9).
