# Supplementary material for: Time trends, factors associated with, and reasons for COVID-19 vaccine hesitancy: A massive online survey of US adults from January-May 2021
Source: PLoS One. 2021 Dec 21;16(12):e0260731. doi: 10.1371/journal.pone.0260731 (PMC8691631; doi:10.1371/journal.pone.0260731)
Supplement: S2 Table — The distribution of responses is provided including and excluding missing responses for each item. (PDF) [file pone.0260731.s003.pdf]

**sTable 2.** Weighted demographics of the report sample by month. The distribution of responses is provided including and excluding missing responses for each item.

| | January | | February | | March | | April | | May | |
|---|---|---|---|---|---|---|---|---|---|---|
| | Full sample | Not missing | Full sample | Not missing | Full sample | Not missing | Full sample | Not missing | Full sample | Not missing |
| **Gender** | | | | | | | | | | |
| Male | 40.04 | 46.03 | 39.34 | 46.09 | 38.79 | 46.01 | 38.93 | 45.94 | 38.76 | 45.68 |
| Female | 46.19 | 53.11 | 45.25 | 53.02 | 44.75 | 53.09 | 44.96 | 53.06 | 45.13 | 53.18 |
| Non-binary | 0.75 | 0.86 | 0.76 | 0.89 | 0.76 | 0.90 | 0.84 | 1.00 | 0.97 | 1.14 |
| Missing | 13.03 | NA | 14.65 | NA | 15.70 | NA | 15.26 | NA | 15.14 | NA |
| **Age group** | | | | | | | | | | |
| 18-24 years | 9.03 | 10.26 | 8.73 | 10.13 | 8.46 | 9.94 | 8.40 | 9.82 | 8.46 | 9.87 |
| 25-34 years | 14.16 | 16.08 | 13.64 | 15.84 | 13.28 | 15.60 | 13.37 | 15.62 | 13.56 | 15.82 |
| 35-44 years | 14.42 | 16.39 | 14.21 | 16.50 | 14.02 | 16.47 | 14.18 | 16.57 | 14.13 | 16.49 |
| 45-54 years | 15.40 | 17.50 | 14.98 | 17.39 | 14.72 | 17.29 | 14.88 | 17.39 | 14.72 | 17.17 |
| 55-64 years | 15.65 | 17.79 | 15.41 | 17.89 | 15.43 | 18.12 | 15.64 | 18.28 | 15.81 | 18.45 |
| 65-74 years | 13.48 | 15.31 | 13.34 | 15.48 | 13.49 | 15.85 | 13.40 | 15.66 | 13.24 | 15.46 |
| ≥ 75 years | 5.87 | 6.67 | 5.83 | 6.77 | 5.72 | 6.72 | 5.70 | 6.66 | 5.78 | 6.75 |
| Missing | 11.99 | NA | 13.86 | NA | 14.88 | NA | 14.44 | NA | 14.31 | NA |
| **Race/ethnicity** | | | | | | | | | | |
| White | 59.70 | 69.21 | 58.49 | 69.39 | 57.63 | 69.16 | 57.84 | 69.08 | 57.87 | 69.07 |
| Hispanic | 14.68 | 17.02 | 14.14 | 16.78 | 13.82 | 16.59 | 13.90 | 16.60 | 13.77 | 16.44 |
| Black | 6.33 | 7.34 | 6.10 | 7.23 | 5.76 | 6.91 | 5.54 | 6.62 | 5.46 | 6.52 |
| Asian | 2.42 | 2.80 | 2.46 | 2.92 | 2.51 | 3.01 | 2.84 | 3.39 | 3.01 | 3.59 |
| Native American | 0.92 | 1.07 | 0.89 | 1.05 | 0.75 | 0.90 | 0.71 | 0.84 | 0.71 | 0.84 |
| Pacific Islander | 0.23 | 0.26 | 0.23 | 0.27 | 0.21 | 0.26 | 0.21 | 0.25 | 0.21 | 0.24 |
| Multi-racial | 1.98 | 2.30 | 1.99 | 2.36 | 2.65 | 3.17 | 2.69 | 3.22 | 2.76 | 3.30 |
| Unknown (other or missing) | 13.74 | NA | 15.71 | NA | 16.67 | NA | 16.28 | NA | 16.22 | NA |

Continued on next page

| | | | | | | | | | | |
|---|---|---|---|---|---|---|---|---|---|---|
| **Education level** | | | | | | | | | | |
| ≤ High school | 20.91 | 24.05 | 19.94 | 23.43 | 19.01 | 22.61 | 18.90 | 22.38 | 19.03 | 22.51 |
| Some college | 32.00 | 36.81 | 31.13 | 36.59 | 30.73 | 36.55 | 30.68 | 36.33 | 30.84 | 36.46 |
| 4 year degree | 19.76 | 22.73 | 19.57 | 23.00 | 19.83 | 23.58 | 20.20 | 23.91 | 20.12 | 23.79 |
| Master's | 10.15 | 11.67 | 10.27 | 12.07 | 10.35 | 12.30 | 10.44 | 12.36 | 10.30 | 12.18 |
| Professional (e.g., MD, JD) | 2.50 | 2.87 | 2.53 | 2.98 | 2.53 | 3.01 | 2.54 | 3.01 | 2.57 | 3.03 |
| Doctorate | 1.61 | 1.86 | 1.64 | 1.93 | 1.63 | 1.94 | 1.70 | 2.01 | 1.71 | 2.02 |
| No response | 13.06 | NA | 14.92 | NA | 15.91 | NA | 15.54 | NA | 15.43 | NA |
| **Employment status** | | | | | | | | | | |
| Work outside home | 35.66 | 41.44 | 35.27 | 41.80 | 35.39 | 42.41 | 35.59 | 42.48 | 35.99 | 42.89 |
| Work at home | 11.81 | 13.72 | 11.06 | 13.11 | 10.82 | 12.97 | 10.95 | 13.07 | 10.61 | 12.65 |
| Does not work for pay | 38.59 | 44.84 | 38.04 | 45.09 | 37.23 | 44.62 | 37.24 | 44.45 | 37.31 | 44.46 |
| Missing | 13.95 | NA | 15.63 | NA | 16.55 | NA | 16.21 | NA | 16.09 | NA |
