# Supplementary material for: Time trends, factors associated with, and reasons for COVID-19 vaccine hesitancy: A massive online survey of US adults from January-May 2021
Source: PLoS One. 2021 Dec 21;16(12):e0260731. doi: 10.1371/journal.pone.0260731 (PMC8691631; doi:10.1371/journal.pone.0260731)
Supplement: S3 Table — (PDF) [file pone.0260731.s004.pdf]

sTable 3. COVID-19 vaccine receipt and intent by month (January-May, 2021), among US adults

	January	February	March	April	May	Difference (May - January)
Already vaccinated	10.9 (10.8, 10.9)	24.3 (24.2, 24.4)	44.7 (44.6, 44.8)	68.3 (68.1, 68.4)	76.4 (76.2, 76.6)	65.5 (65.4, 65.7)
Yes, definitely	45.4 (45.3, 45.5)	37.4 (37.3, 37.5)	24.3 (24.2, 24.4)	8.1 (8.0, 8.2)	3.3 (3.2, 3.4)	-42.1 (-42.2, -42.0)
Yes, probably	18.3 (18.2, 18.4)	14.6 (14.5, 14.7)	10.6 (10.5, 10.7)	5.9 (5.8, 6.0)	3.7 (3.7, 3.8)	-14.6 (-14.7, -14.5)
No, probably not	13.0 (13.0, 13.1)	11.5 (11.5, 11.6)	9.3 (9.2, 9.3)	7.1 (7.0, 7.1)	6.0 (5.9, 6.1)	-7.1 (-7.2, -6.9)
No, definitely not	12.4 (12.3, 12.4)	12.1 (12.1, 12.2)	11.0 (11.0, 11.1)	10.7 (10.6, 10.7)	10.6 (10.5, 10.7)	-1.8 (-1.9, -1.7)
Hesitant[a]	25.4 (25.3, 25.5)	23.7 (23.6, 23.8)	20.3 (20.2, 20.4)	17.7 (17.6, 17.8)	16.6 (16.4, 16.7)	-8.9 (-9.0, -8.7)

[a] Answered that they probably or definitely would not choose to get vaccinated if offered a vaccine to prevent COVID-19
