# Supplementary material for: Time trends, factors associated with, and reasons for COVID-19 vaccine hesitancy: A massive online survey of US adults from January-May 2021
Source: PLoS One. 2021 Dec 21;16(12):e0260731. doi: 10.1371/journal.pone.0260731 (PMC8691631; doi:10.1371/journal.pone.0260731)
Supplement: S4 Table — (PDF) [file pone.0260731.s005.pdf]

sTable 4. COVID-19 vaccine hesitancy by race/ethnicity, education level, US region and county Trump vote share in the 2020 presidential election, respectively, by month (January-May, 2021), among US adults

	January	February	March	April	May	Difference (May - January)
	% (95% CI)					
Race/ethnicity among 18 - 34 year olds[a]						
White	31.0 (30.7, 31.3)	29.7 (29.4, 30.0)	27.2 (26.8, 27.5)	23.9 (23.6, 24.3)	22.5 (22.0, 23.0)	-8.5 (-9.1, -7.9)
Hispanic	31.7 (31.2, 32.3)	28.5 (27.9, 29.1)	23.6 (23.0, 24.2)	19.0 (18.4, 19.7)	16.3 (15.5, 17.1)	-15.4 (-16.4, -14.4)
Black	58.9 (57.7, 60.0)	53.0 (51.8, 54.3)	43.1 (41.8, 44.5)	34.0 (32.5, 35.6)	27.4 (25.3, 29.4)	-31.5 (-33.8, -29.2)
Asian	12.4 (11.4, 13.4)	11.6 (10.6, 12.7)	8.0 (7.1, 8.9)	4.6 (3.9, 5.2)	3.2 (2.4, 4.0)	-9.2 (-10.5, -7.9)
Native American	43.2 (40.4, 46.0)	39.4 (36.4, 42.4)	32.4 (29.2, 35.7)	30.8 (27.3, 34.4)	33.7 (28.1, 39.3)	-9.4 (-15.7, -3.2)
Pacific Islander	43.0 (37.0, 49.0)	30.5 (24.9, 36.1)	30.8 (24.3, 37.4)	27.0 (18.4, 35.5)	15.8 (7.9, 23.6)	-27.2 (-37.1, -17.4)
Multi-racial	38.2 (36.9, 39.6)	37.6 (36.2, 39.1)	35.5 (34.1, 36.8)	31.2 (29.7, 32.7)	29.0 (26.9, 31.1)	-9.2 (-11.7, -6.7)
Education level						
≤ High school	34.8 (34.6, 35.1)	31.9 (31.6, 32.2)	26.5 (26.2, 26.7)	22.9 (22.6, 23.2)	20.3 (20.0, 20.7)	-14.5 (-14.9, -14.0)
Some college	27.2 (27.0, 27.3)	25.3 (25.1, 25.5)	21.6 (21.5, 21.8)	18.9 (18.7, 19.1)	17.9 (17.7, 18.2)	-9.2 (-9.5, -9.0)
4 year degree	15.2 (15.1, 15.4)	14.3 (14.2, 14.5)	12.2 (12.0, 12.4)	10.8 (10.7, 11.0)	10.8 (10.5, 11.0)	-4.5 (-4.8, -4.2)
Master's	11.5 (11.4, 11.7)	10.5 (10.3, 10.7)	9.0 (8.8, 9.2)	8.0 (7.8, 8.2)	7.8 (7.6, 8.1)	-3.7 (-4.0, -3.4)
Professional (e.g., MD, JD)	11.9 (11.4, 12.3)	11.8 (11.3, 12.2)	11.0 (10.6, 11.4)	10.0 (9.5, 10.4)	11.1 (10.4, 11.8)	-0.8 (-1.6, 0.0)
Doctorate	16.5 (15.8, 17.1)	16.1 (15.4, 16.8)	14.6 (13.9, 15.2)	14.8 (14.1, 15.5)	14.6 (13.5, 15.6)	-1.9 (-3.1, -0.7)
US Region						
Midwest	26.3 (26.1, 26.5)	24.7 (24.5, 24.9)	21.6 (21.5, 21.8)	19.0 (18.8, 19.2)	17.7 (17.4, 17.9)	-8.6 (-9.0, -8.3)
South	28.5 (28.3, 28.7)	26.8 (26.6, 26.9)	22.9 (22.7, 23.1)	20.0 (19.8, 20.2)	18.8 (18.6, 19.1)	-9.7 (-10.0, -9.4)
Pacific	20.0 (19.7, 20.2)	18.0 (17.8, 18.3)	15.2 (15.0, 15.4)	12.6 (12.3, 12.8)	11.7 (11.3, 12.0)	-8.3 (-8.7, -7.9)
Mountain	24.4 (24.0, 24.7)	22.9 (22.6, 23.3)	20.3 (19.9, 20.6)	18.2 (17.9, 18.6)	17.5 (17.0, 18.0)	-6.8 (-7.4, -6.2)
Northeast	21.6 (21.4, 21.8)	19.6 (19.4, 19.8)	16.1 (15.8, 16.3)	13.7 (13.5, 13.9)	12.2 (11.9, 12.5)	-9.4 (-9.7, -9.0)
County Trump vote share						
Lowest quartile	21.7 (21.6, 21.8)	19.7 (19.6, 19.8)	16.3 (16.2, 16.4)	13.7 (13.6, 13.8)	12.4 (12.3, 12.6)	-9.3 (-9.5, -9.1)
Second lowest quartile	29.7 (29.5, 29.9)	28.4 (28.2, 28.7)	25.2 (24.9, 25.4)	22.5 (22.3, 22.7)	21.4 (21.1, 21.8)	-8.3 (-8.7, -7.9)
Second highest quartile	35.1 (34.7, 35.4)	33.6 (33.3, 34.0)	30.2 (29.9, 30.5)	27.9 (27.5, 28.3)	27.0 (26.5, 27.6)	-8.1 (-8.7, -7.4)
Highest quartile	38.8 (38.2, 39.3)	38.4 (37.8, 38.9)	35.4 (34.8, 35.9)	32.6 (32.0, 33.2)	31.9 (31.1, 32.8)	-6.8 (-7.8, -5.8)

Juris Doctorate= JD; MD=Doctor of Medicine.

[a] Race/ethnicity categories are reported among young adults (18-34 years) only, because there was an interaction between race/ethnicity and age, such that race/ethnic comparisons differed by age group. Younger adults were selected because hesitancy was higher among younger versus older adults. Race/ethnicity groups other than the group labeled "Hispanic" are non-Hispanic.
