# Supplementary material for: Time trends, factors associated with, and reasons for COVID-19 vaccine hesitancy: A massive online survey of US adults from January-May 2021
Source: PLoS One. 2021 Dec 21;16(12):e0260731. doi: 10.1371/journal.pone.0260731 (PMC8691631; doi:10.1371/journal.pone.0260731)
Supplement: S5 Table — (PDF) [file pone.0260731.s006.pdf]

sTable 5. COVID-19 vaccine hesitancy in May 2021 by age groups, stratified by race/ethnicity[a] among US adults

	Sample		COVID-19 vaccine hesitant		
	n	%	% (95% CI)	RR (95% CI)	Adj. RR (95% CI)
White					
18-24 years	9858	1.9	23.5 (22.5, 24.6)	2.89 (2.75, 3.04)	1.46 (1.38, 1.53)
25-34 years	35244	6.7	21.9 (21.3, 22.4)	2.69 (2.60, 2.79)	1.64 (1.57, 1.70)
35-44 years	49337	9.4	18.6 (18.2, 19.0)	2.29 (2.21, 2.37)	1.47 (1.42, 1.53)
45-54 years	54805	10.4	18.2 (17.9, 18.6)	2.24 (2.17, 2.32)	1.42 (1.37, 1.48)
55-64 years	74941	14.3	13.3 (13.0, 13.6)	1.64 (1.58, 1.69)	1.21 (1.16, 1.25)
65-74 years	76794	14.6	8.1 (7.9, 8.3)	1.00 (NA)	1.00 (NA)
≥ 75 years	36019	6.9	6.0 (5.7, 6.2)	0.73 (0.69, 0.77)	0.76 (0.73, 0.80)
Hispanic					
18-24 years	3043	0.6	17.1 (15.5, 18.7)	2.84 (2.44, 3.24)	1.64 (1.42, 1.86)
25-34 years	8668	1.6	15.7 (14.8, 16.6)	2.61 (2.30, 2.92)	1.74 (1.54, 1.93)
35-44 years	11700	2.2	12.5 (11.7, 13.3)	2.08 (1.82, 2.34)	1.42 (1.25, 1.59)
45-54 years	12943	2.5	9.0 (8.5, 9.6)	1.50 (1.32, 1.69)	1.13 (1.00, 1.26)
55-64 years	11507	2.2	7.7 (7.1, 8.2)	1.27 (1.11, 1.44)	1.05 (0.93, 1.18)
65-74 years	6481	1.2	6.0 (5.4, 6.7)	1.00 (NA)	1.00 (NA)
≥ 75 years	1921	0.4	15.2 (13.1, 17.4)	2.53 (2.08, 2.97)	1.61 (1.39, 1.84)
Black					
18-24 years	626	0.1	30.9 (26.6, 35.2)	7.26 (5.92, 8.61)	3.51 (2.77, 4.24)
25-34 years	2205	0.4	25.3 (23.3, 27.4)	5.96 (5.09, 6.83)	3.73 (3.18, 4.28)
35-44 years	3785	0.7	18.7 (17.3, 20.0)	4.39 (3.77, 5.01)	2.96 (2.54, 3.37)
45-54 years	5536	1.1	12.2 (11.2, 13.1)	2.86 (2.45, 3.28)	2.13 (1.83, 2.44)
55-64 years	8289	1.6	7.8 (7.2, 8.5)	1.84 (1.57, 2.11)	1.47 (1.26, 1.69)
65-74 years	6390	1.2	4.3 (3.7, 4.8)	1.00 (NA)	1.00 (NA)
≥ 75 years	1669	0.3	3.0 (2.1, 4.0)	0.71 (0.47, 0.95)	0.75 (0.51, 0.98)
Asian					
18-24 years	612	0.1	4.0 (2.1, 6.0)	2.45 (0.96, 3.94)	1.68 (0.68, 2.68)
25-34 years	2288	0.4	2.7 (2.0, 3.4)	1.65 (0.90, 2.41)	1.42 (0.78, 2.06)
35-44 years	2745	0.5	2.9 (2.2, 3.6)	1.75 (0.96, 2.55)	1.56 (0.87, 2.24)
45-54 years	2234	0.4	3.3 (2.4, 4.2)	2.02 (1.08, 2.95)	1.70 (0.93, 2.47)
55-64 years	1957	0.4	2.8 (2.0, 3.6)	1.69 (0.88, 2.49)	1.50 (0.80, 2.21)
65-74 years	1527	0.3	1.7 (1.0, 2.3)	1.00 (NA)	1.00 (NA)
≥ 75 years	586	0.1	2.4 (0.9, 3.9)	1.48 (0.41, 2.54)	1.51 (0.44, 2.58)
Native American					
18-24 years	142	0.0	37.3 (26.0, 48.7)	2.76 (1.73, 3.79)	1.60 (0.95, 2.24)
25-34 years	406	0.1	31.3 (25.8, 36.7)	2.31 (1.66, 2.96)	1.28 (0.98, 1.58)
35-44 years	574	0.1	27.2 (23.1, 31.4)	2.01 (1.48, 2.55)	1.32 (1.03, 1.61)
45-54 years	841	0.2	24.3 (21.1, 27.6)	1.80 (1.34, 2.26)	1.19 (0.94, 1.45)
55-64 years	1031	0.2	21.8 (19.0, 24.6)	1.61 (1.20, 2.02)	1.18 (0.93, 1.43)
65-74 years	691	0.1	13.5 (10.6, 16.5)	1.00 (NA)	1.00 (NA)
≥ 75 years	244	0.0	17.7 (12.1, 23.3)	1.31 (0.80, 1.81)	1.36 (0.92, 1.81)

Continued next page

Pacific Islander[a]					
18-24 years	20	0.0	6.0 (-4.3, 16.2)	0.81 (-0.66, 2.28)	0.62 (-0.45, 1.69)
25-34 years	68	0.0	21.2 (11.2, 31.1)	2.87 (0.77, 4.97)	1.47 (0.46, 2.47)
35-44 years	150	0.0	14.1 (7.9, 20.4)	1.92 (0.55, 3.29)	1.46 (0.50, 2.43)
45-54 years	230	0.0	16.5 (10.8, 22.1)	2.24 (0.77, 3.70)	1.54 (0.59, 2.48)
55-64 years	277	0.1	11.6 (7.2, 16.1)	1.58 (0.51, 2.65)	1.28 (0.46, 2.09)
65-74 years	190	0.0	7.4 (3.3, 11.5)	1.00 (NA)	1.00 (NA)
≥ 75 years	56	0.0	14.5 (2.6, 26.4)	1.97 (0.01, 3.92)	1.64 (0.44, 2.83)
Multi-racial					
18-24 years	880	0.2	29.7 (26.0, 33.4)	1.90 (1.58, 2.23)	1.20 (1.03, 1.38)
25-34 years	2209	0.4	28.5 (26.3, 30.7)	1.83 (1.57, 2.08)	1.27 (1.11, 1.42)
35-44 years	2557	0.5	30.0 (28.0, 32.0)	1.92 (1.67, 2.18)	1.29 (1.13, 1.44)
45-54 years	2385	0.5	28.3 (26.3, 30.4)	1.82 (1.57, 2.07)	1.23 (1.08, 1.38)
55-64 years	2491	0.5	24.6 (22.7, 26.5)	1.58 (1.36, 1.80)	1.16 (1.02, 1.30)
65-74 years	1803	0.3	15.6 (13.8, 17.4)	1.00 (NA)	1.00 (NA)
≥ 75 years	660	0.1	17.6 (13.6, 21.6)	1.13 (0.84, 1.42)	0.89 (0.72, 1.05)

[a] Race/ethnicity groups other than the group labeled "Hispanic" are non-Hispanic.
[b] The Hispanic ≥ 75 years group has unusually high rates of self-describe gender; results were attenuated in a sensitivity analysis with that group (self-describe gender) removed.
[c] Due to the limited sample size of age groups among Pacific Islanders, the 95% CI of the RR estimates are large.
