# Supplementary material for: Time trends, factors associated with, and reasons for COVID-19 vaccine hesitancy: A massive online survey of US adults from January-May 2021
Source: PLoS One. 2021 Dec 21;16(12):e0260731. doi: 10.1371/journal.pone.0260731 (PMC8691631; doi:10.1371/journal.pone.0260731)
Supplement: S6 Table — (PDF) [file pone.0260731.s007.pdf]

sTable 6. COVID-19 vaccine hesitancy in May 2021 by race/ethnicity[a], stratified by age groups, among US adults

	Sample		COVID-19 vaccine hesitant		
	**n**	**%**	**% (95% CI)**	**RR (95% CI)**	**Adj. RR (95% CI)**
18-24 years					
White	9858	1.9	23.5 (22.5, 24.6)	1.00 (NA)	1.00 (NA)
Hispanic	3043	0.6	17.1 (15.5, 18.7)	0.73 (0.65, 0.80)	0.92 (0.84, 1.01)
Black	626	0.1	30.9 (26.6, 35.2)	1.31 (1.12, 1.51)	1.47 (1.21, 1.73)
Asian	612	0.1	4.0 (2.1, 6.0)	0.17 (0.09, 0.25)	0.38 (0.20, 0.55)
Native American	142	0.0	37.3 (26.0, 48.7)	1.59 (1.10, 2.08)	1.43 (0.92, 1.95)
Pacific Islander	20	0.0	6.0 (-4.3, 16.2)	0.25 (-0.18, 0.69)	0.44 (-0.29, 1.17)
Multi-racial	880	0.2	29.7 (26.0, 33.4)	1.26 (1.09, 1.43)	1.26 (1.12, 1.39)
25-34 years					
White	35244	6.7	21.9 (21.3, 22.4)	1.00 (NA)	1.00 (NA)
Hispanic	8668	1.6	15.7 (14.8, 16.6)	0.72 (0.68, 0.76)	0.87 (0.82, 0.92)
Black	2205	0.4	25.3 (23.3, 27.4)	1.16 (1.06, 1.26)	1.38 (1.26, 1.50)
Asian	2288	0.4	2.7 (2.0, 3.4)	0.12 (0.09, 0.16)	0.28 (0.21, 0.36)
Native American	406	0.1	31.3 (25.8, 36.7)	1.43 (1.18, 1.68)	1.02 (0.87, 1.18)
Pacific Islander	68	0.0	21.2 (11.2, 31.1)	0.97 (0.51, 1.43)	0.93 (0.52, 1.35)
Multi-racial	2209	0.4	28.5 (26.3, 30.7)	1.30 (1.20, 1.41)	1.18 (1.09, 1.26)
35-44 years					
White	49337	9.4	18.6 (18.2, 19.0)	1.00 (NA)	1.00 (NA)
Hispanic	11700	2.2	12.5 (11.7, 13.3)	0.67 (0.63, 0.72)	0.79 (0.74, 0.84)
Black	3785	0.7	18.7 (17.3, 20.0)	1.00 (0.93, 1.08)	1.22 (1.13, 1.32)
Asian	2745	0.5	2.9 (2.2, 3.6)	0.16 (0.12, 0.20)	0.34 (0.26, 0.43)
Native American	574	0.1	27.2 (23.1, 31.4)	1.46 (1.24, 1.69)	1.17 (1.03, 1.32)
Pacific Islander	150	0.0	14.1 (7.9, 20.4)	0.76 (0.42, 1.10)	1.04 (0.62, 1.46)
Multi-racial	2557	0.5	30.0 (28.0, 32.0)	1.61 (1.50, 1.73)	1.33 (1.25, 1.41)
45-54 years					
White	54805	10.4	18.2 (17.9, 18.6)	1.00 (NA)	1.00 (NA)
Hispanic	12943	2.5	9.0 (8.5, 9.6)	0.50 (0.47, 0.53)	0.65 (0.61, 0.69)
Black	5536	1.1	12.2 (11.2, 13.1)	0.67 (0.61, 0.72)	0.91 (0.84, 0.98)
Asian	2234	0.4	3.3 (2.4, 4.2)	0.18 (0.13, 0.23)	0.39 (0.29, 0.49)
Native American	841	0.2	24.3 (21.1, 27.6)	1.33 (1.16, 1.51)	1.10 (0.97, 1.23)
Pacific Islander	230	0.0	16.5 (10.8, 22.1)	0.90 (0.59, 1.21)	1.13 (0.76, 1.49)
Multi-racial	2385	0.5	28.3 (26.3, 30.4)	1.55 (1.44, 1.67)	1.32 (1.23, 1.40)
55-64 years					
White	74941	14.3	13.3 (13.0, 13.6)	1.00 (NA)	1.00 (NA)
Hispanic	11507	2.2	7.7 (7.1, 8.2)	0.58 (0.53, 0.62)	0.71 (0.66, 0.76)
Black	8289	1.6	7.8 (7.2, 8.5)	0.59 (0.54, 0.64)	0.74 (0.68, 0.81)
Asian	1957	0.4	2.8 (2.0, 3.6)	0.21 (0.15, 0.27)	0.41 (0.29, 0.52)
Native American	1031	0.2	21.8 (19.0, 24.6)	1.64 (1.42, 1.85)	1.27 (1.14, 1.41)
Pacific Islander	277	0.1	11.6 (7.2, 16.1)	0.88 (0.54, 1.21)	1.10 (0.69, 1.51)
Multi-racial	2491	0.5	24.6 (22.7, 26.5)	1.85 (1.71, 2.00)	1.46 (1.37, 1.56)

Continued next page

65-74 years					
White	76794	14.6	8.1 (7.9, 8.3)	1.00 (NA)	1.00 (NA)
Hispanic	6481	1.2	6.0 (5.4, 6.7)	0.74 (0.66, 0.82)	0.82 (0.74, 0.90)
Black	6390	1.2	4.3 (3.7, 4.8)	0.52 (0.46, 0.59)	0.61 (0.53, 0.68)
Asian	1527	0.3	1.7 (1.0, 2.3)	0.20 (0.13, 0.28)	0.33 (0.21, 0.45)
Native American	691	0.1	13.5 (10.6, 16.5)	1.67 (1.30, 2.03)	1.31 (1.07, 1.55)
Pacific Islander	190	0.0	7.4 (3.3, 11.5)	0.91 (0.40, 1.41)	1.04 (0.50, 1.59)
Multi-racial	1803	0.3	15.6 (13.8, 17.4)	1.92 (1.69, 2.15)	1.52 (1.36, 1.68)
≥ 75 years					
White	36019	6.9	6.0 (5.7, 6.2)	1.00 (NA)	1.00 (NA)
Hispanic	1921	0.4	15.2 (13.1, 17.4)	2.56 (2.18, 2.94)	1.73 (1.54, 1.92)
Black	1669	0.3	3.0 (2.1, 4.0)	0.50 (0.34, 0.67)	0.59 (0.42, 0.77)
Asian	586	0.1	2.4 (0.9, 3.9)	0.41 (0.16, 0.66)	0.65 (0.26, 1.03)
Native American	244	0.0	17.7 (12.1, 23.3)	2.97 (2.02, 3.91)	2.33 (1.69, 2.98)
Pacific Islander	56	0.0	14.5 (2.6, 26.4)	2.43 (0.43, 4.43)	2.23 (1.09, 3.38)
Multi-racial	660	0.1	17.6 (13.6, 21.6)	2.95 (2.27, 3.64)	1.77 (1.48, 2.06)

[a] Race/ethnicity groups other than the group labeled “Hispanic” are non-Hispanic.

[b] The Hispanic ≥ 75 years has unusually high rates of self-describe gender; results were attenuated in a sensitivity analysis with that group (self-describe gender) removed
