# Supplementary material for: Time trends, factors associated with, and reasons for COVID-19 vaccine hesitancy: A massive online survey of US adults from January-May 2021
Source: PLoS One. 2021 Dec 21;16(12):e0260731. doi: 10.1371/journal.pone.0260731 (PMC8691631; doi:10.1371/journal.pone.0260731)
Supplement: S7 Table — (PDF) [file pone.0260731.s008.pdf]

sTable 7. COVID-19 vaccine hesitancy in May 2021 by health condition and status[a] among US adults

	Sample		COVID-19 vaccine hesitant		
	**n**	**%**	**% (95% CI)**	**RR (95% CI)**	**Adj. RR (95% CI)**
None	182782	34.8	18.7 (18.5, 19.0)	1.0 (NA)	1.0 (NA)
Cancer (other than skin)	10355	2.0	8.9 (8.2, 9.5)	0.47 (0.44, 0.51)	0.80 (0.75, 0.85)
Diabetes Type II	19671	3.7	9.6 (9.1, 10.1)	0.51 (0.49, 0.54)	0.83 (0.79, 0.86)
Obesity	50985	9.7	10.9 (10.5, 11.3)	0.58 (0.56, 0.60)	0.79 (0.76, 0.81)
Diabetes Type I	2614	0.5	12.2 (10.7, 13.8)	0.65 (0.57, 0.74)	0.86 (0.75, 0.96)
High blood pressure	48953	9.3	13.2 (12.8, 13.6)	0.70 (0.68, 0.73)	0.95 (0.92, 0.98)
Kidney disease	2344	0.4	13.3 (11.6, 15.0)	0.71 (0.62, 0.80)	1.07 (0.94, 1.19)
Chronic obstructive pulmonary disease	6380	1.2	13.7 (12.5, 15.0)	0.73 (0.67, 0.80)	1.03 (0.96, 1.11)
Multiple conditions	127693	24.3	14.0 (13.7, 14.2)	0.75 (0.73, 0.76)	1.13 (1.11, 1.15)
Heart attack, heart disease, or other heart condition	12821	2.4	14.6 (13.9, 15.4)	0.78 (0.74, 0.83)	1.11 (1.06, 1.17)
Autoimmune disorder	9700	1.8	15.1 (14.2, 16.0)	0.80 (0.76, 0.85)	1.06 (1.01, 1.12)
Asthma	26313	5.0	16.6 (15.9, 17.2)	0.89 (0.85, 0.92)	1.03 (0.99, 1.06)
Weakened or compromised immune system	4359	0.8	20.7 (19.1, 22.2)	1.10 (1.02, 1.19)	1.43 (1.33, 1.52)
No response	20674	3.9	35.5 (34.6, 36.3)	1.89 (1.84, 1.95)	1.71 (1.66, 1.76)

[a] Participants were categorized as having high blood pressure only, each of the other conditions with or without high blood pressure, or "multiple conditions," defined as at least two conditions excluding high blood pressure, which was relatively common and has limited support as a risk-factor for poor COVID-19 outcomes.
