# Supplementary material for: Time trends, factors associated with, and reasons for COVID-19 vaccine hesitancy: A massive online survey of US adults from January-May 2021
Source: PLoS One. 2021 Dec 21;16(12):e0260731. doi: 10.1371/journal.pone.0260731 (PMC8691631; doi:10.1371/journal.pone.0260731)
Supplement: S8 Table — (PDF) [file pone.0260731.s009.pdf]

**sTable 8.** Sensitivity analysis: COVID-19 vaccine hesitancy in May 2021 by demographics among US adults, including those that self-described gender (N=529,658)

| | Sample | | COVID-19 vaccine hesitant | | |
|---|---|---|---|---|---|
| | **n** | **%** | **% (95% CI)** | **RR (95% CI)** | **Adj. RR (95% CI)** |
| Gender | | | | | |
| Male | 159427 | 30.1 | 16.6 (16.4, 16.9) | 1.0 (NA) | 1.0 (NA) |
| Female | 294983 | 55.7 | 13.2 (13.1, 13.4) | 0.79 (0.78, 0.81) | 1.12 (1.10, 1.14) |
| Non-binary | 3232 | 0.6 | 18.2 (16.1, 20.3) | 1.10 (0.97, 1.22) | 0.99 (0.88, 1.10) |
| Self-described | 4014 | 0.8 | 64.2 (62.3, 66.1) | 3.86 (3.73, 3.99) | 1.42 (1.37, 1.47) |
| Missing | 68002 | 12.8 | 26.3 (25.8, 26.7) | 1.58 (1.54, 1.61) | 1.39 (1.34, 1.44) |
| Age group | | | | | |
| 18-24 years | 15678 | 3.0 | 22.9 (22.1, 23.7) | 2.79 (2.67, 2.91) | [b] |
| 25-34 years | 52640 | 9.9 | 21.3 (20.8, 21.7) | 2.60 (2.52, 2.68) | |
| 35-44 years | 73245 | 13.8 | 18.4 (18.1, 18.8) | 2.25 (2.18, 2.32) | |
| 45-54 years | 81578 | 15.4 | 17.0 (16.7, 17.3) | 2.07 (2.01, 2.13) | |
| 55-64 years | 103380 | 19.5 | 12.9 (12.7, 13.1) | 1.57 (1.53, 1.62) | |
| 65-74 years | 95964 | 18.1 | 8.2 (8.0, 8.4) | 1.0 (NA) | |
| ≥ 75 years | 42657 | 8.1 | 9.8 (9.4, 10.2) | 1.20 (1.14, 1.25) | |
| Missing | 64516 | 12.2 | 24.6 (24.1, 25.0) | 3.00 (2.91, 3.09) | |
| Race/ethnicity | | | | | |
| White | 338578 | 63.9 | 15.8 (15.6, 16.0) | 1.0 (NA) | [b] |
| Hispanic | 57608 | 10.9 | 13.4 (13.0, 13.7) | 0.85 (0.82, 0.87) | |
| Black | 28625 | 5.4 | 13.0 (12.5, 13.5) | 0.82 (0.79, 0.86) | |
| Asian | 12012 | 2.3 | 3.2 (2.8, 3.6) | 0.20 (0.17, 0.23) | |
| Native American | 3993 | 0.8 | 25.3 (23.4, 27.2) | 1.60 (1.48, 1.72) | |
| Pacific Islander | 1002 | 0.2 | 13.9 (11.3, 16.5) | 0.88 (0.71, 1.04) | |
| Multi-racial | 13433 | 2.5 | 29.2 (28.2, 30.2) | 1.85 (1.78, 1.92) | |
| Missing | 74407 | 14.0 | 26.5 (26.1, 27.0) | 1.68 (1.65, 1.71) | |
| Education level | | | | | |
| ≤ High school | 92557 | 17.5 | 20.8 (20.4, 21.1) | 1.88 (1.83, 1.93) | 1.56 (1.52, 1.60) |
| Some college | 167096 | 31.5 | 18.3 (18.1, 18.6) | 1.66 (1.62, 1.70) | 1.37 (1.34, 1.40) |
| 4 year degree | 110944 | 20.9 | 11.0 (10.8, 11.3) | 1.0 (NA) | 1.0 (NA) |
| Master's | 62862 | 11.9 | 8.3 (8.1, 8.6) | 0.75 (0.72, 0.78) | 0.90 (0.87, 0.92) |
| Professional (e.g., MD, JD) | 14970 | 2.8 | 12.3 (11.6, 13.0) | 1.12 (1.05, 1.18) | 1.09 (1.04, 1.15) |
| Doctorate | 10969 | 2.1 | 23.9 (22.7, 25.1) | 2.16 (2.05, 2.28) | 1.20 (1.14, 1.25) |
| Missing | 70260 | 13.3 | 23.9 (23.5, 24.3) | 2.16 (2.10, 2.22) | 1.18 (1.10, 1.25) |
| Employment status | | | | | |
| Work outside home | 176197 | 33.3 | 21.2 (20.9, 21.4) | 2.48 (2.39, 2.57) | 1.33 (1.28, 1.37) |
| Work at home | 57246 | 10.8 | 8.5 (8.2, 8.8) | 1.0 (NA) | 1.0 (NA) |
| Does not work for pay | 223071 | 42.1 | 12.7 (12.5, 12.9) | 1.49 (1.43, 1.54) | 1.34 (1.29, 1.38) |
| Missing | 73144 | 13.8 | 23.9 (23.5, 24.3) | 2.80 (2.69, 2.91) | 1.33 (1.25, 1.41) |

Continued on next page

| | | | | | |
|---|---|---|---|---|---|
| US Region | | | | | |
| Midwest | 126686 | 23.9 | 18.1 (17.9, 18.4) | 1.50 (1.46, 1.55) | 1.10 (1.07, 1.13) |
| South | 182852 | 34.5 | 19.2 (19.0, 19.5) | 1.59 (1.55, 1.64) | 1.13 (1.10, 1.16) |
| Pacific | 73521 | 13.9 | 12.1 (11.7, 12.4) | 1.0 (NA) | 1.0 (NA) |
| Mountain | 42261 | 8.0 | 17.9 (17.4, 18.5) | 1.49 (1.43, 1.55) | 1.11 (1.07, 1.15) |
| Northeast | 88229 | 16.7 | 12.6 (12.3, 12.9) | 1.04 (1.01, 1.08) | 0.96 (0.93, 0.99) |
| Territories | 191 | <0.05 | 12.0 (6.3, 17.8) | 1.00 (0.52, 1.48) | 0.64 (0.44, 0.84) |
| Missing | 15918 | 3.0 | 33.3 (32.3, 34.3) | 2.76 (2.64, 2.87) | [c] |
| County urban classification | | | | | |
| Large central metro | 120722 | 22.8 | 11.7 (11.5, 12.0) | 1.0 (NA) | 1.0 (NA) |
| Large fringe metro | 115854 | 21.9 | 14.3 (14.0, 14.5) | 1.22 (1.18, 1.25) | 1.03 (1.01, 1.06) |
| Medium metro | 138457 | 26.1 | 16.8 (16.5, 17.1) | 1.43 (1.39, 1.47) | 1.13 (1.10, 1.16) |
| Small metro | 57778 | 10.9 | 21.0 (20.6, 21.5) | 1.79 (1.74, 1.85) | 1.18 (1.15, 1.22) |
| Micropolitan | 49266 | 9.3 | 24.2 (23.7, 24.7) | 2.06 (2.00, 2.12) | 1.19 (1.15, 1.23) |
| Non-core | 31472 | 5.9 | 27.4 (26.8, 28.1) | 2.34 (2.27, 2.41) | 1.23 (1.19, 1.27) |
| Missing | 16109 | 3.0 | 33.0 (32.0, 34.0) | 2.82 (2.71, 2.92) | [c] |

Juris Doctorate= JD; MD=Doctor of Medicine; NA=not applicable; NH=Non-Hispanic

[a] Race/ethnicity groups other than the group labeled “Hispanic” are non-Hispanic.

[b] Due to an interaction between age group and race/ethnicity, adjusted relative risks from the multivariable model are not reported in this table.

[c] Reliable estimates could not be calculated for the missing category for variables based on participants’ zip code, due to collinearity.
