# Supplementary material for: Time trends, factors associated with, and reasons for COVID-19 vaccine hesitancy: A massive online survey of US adults from January-May 2021
Source: PLoS One. 2021 Dec 21;16(12):e0260731. doi: 10.1371/journal.pone.0260731 (PMC8691631; doi:10.1371/journal.pone.0260731)
Supplement: S9 Table — (PDF) [file pone.0260731.s010.pdf]

sTable 9. Sensitivity analysis: COVID-19 vaccine hesitancy in May 2021 political/by COVID-19 environment, health status, beliefs and behaviors among US adults, including those that self-described gender (N=529,658)

	Sample		COVID-19 vaccine hesitant		
	N	%	% (95% CI)	RR (95% CI)	Adj. RR (95% CI)
State governor's political party					
Democratic	282446	53.3	14.4 (14.2, 14.6)	1.0 (NA)	[a]
Republican	230264	43.5	19.2 (19.0, 19.4)	1.33 (1.31, 1.36)	
Missing	16948	3.2	31.8 (30.8, 32.7)	2.21 (2.13, 2.28)	
County Trump vote total minus Biden vote total in 2020 presidential election					
Lowest quartile	343255	64.8	12.8 (12.6, 12.9)	1.0 (NA)	1.0 (NA)
Second lowest quartile	101627	19.2	21.9 (21.6, 22.3)	1.72 (1.69, 1.75)	1.27 (1.25, 1.30)
Second highest quartile	47422	9.0	27.6 (27.0, 28.1)	2.16 (2.11, 2.21)	1.34 (1.30, 1.37)
Highest quartile	19712	3.7	32.5 (31.7, 33.4)	2.55 (2.48, 2.62)	1.42 (1.38, 1.47)
Missing	17642	3.3	32.4 (31.5, 33.4)	2.54 (2.46, 2.62)	[b]
County COVID-19 April 2021 county death rate					
Lowest quartile	26160	4.9	24.3 (23.7, 25.0)	1.0 (NA)	1.0 (NA)
Second lowest quartile	168948	31.9	16.2 (15.9, 16.4)	0.66 (0.64, 0.69)	0.97 (0.94, 1.00)
Second highest quartile	214630	40.5	15.7 (15.5, 15.9)	0.65 (0.63, 0.67)	1.00 (0.97, 1.03)
Highest quartile	103804	19.6	17.1 (16.8, 17.4)	0.70 (0.68, 0.73)	1.01 (0.98, 1.04)
Missing	16116	3.0	33.0 (32.0, 34.0)	1.36 (1.30, 1.41)	[b]
Ever tested positive for COVID-19					
Yes	55851	10.5	20.7 (20.2, 21.1)	1.24 (1.22, 1.27)	1.10 (1.08, 1.13)
No or unsure	470576	88.8	16.6 (16.5, 16.8)	1.0 (NA)	1.0 (NA)
Missing	3231	0.6	19.6 (17.8, 21.4)	1.18 (1.07, 1.29)	0.94 (0.86, 1.01)
Ever diagnosed with high-risk medical condition					
One or more conditions	324323	61.2	13.8 (13.6, 13.9)	1.0 (NA)	1.0 (NA)
No condition	184503	34.8	19.4 (19.2, 19.7)	1.41 (1.39, 1.43)	1.01 (0.99, 1.02)
Missing	20832	3.9	35.9 (35.0, 36.8)	2.60 (2.53, 2.67)	1.70 (1.65, 1.75)
Extent worried that you or someone in immediate family might become seriously ill from COVID-19					
Worried	209897	39.6	8.8 (8.6, 9.0)	1.0 (NA)	1.0 (NA)
Not too worried	164794	31.1	13.7 (13.5, 13.9)	1.55 (1.52, 1.59)	1.31 (1.28, 1.35)
Not worried at all	98919	18.7	33.7 (33.3, 34.1)	3.82 (3.74, 3.91)	1.78 (1.74, 1.83)
Missing	56048	10.6	24.8 (24.3, 25.3)	2.81 (2.74, 2.89)	1.30 (1.11, 1.49)
Lives with someone or is 65 years or older					
Yes	209290	39.5	12.6 (12.4, 12.8)	1.0 (NA)	1.0 (NA)
No	204999	38.7	18.1 (17.9, 18.3)	1.44 (1.41, 1.46)	1.07 (1.04, 1.09)
No response	115369	21.8	21.2 (20.9, 21.6)	1.68 (1.65, 1.72)	1.11 (1.08, 1.13)
Past-year flu vaccine					
Yes	280787	53.0	5.6 (5.5, 5.7)	1.0 (NA)	1.0 (NA)
No or unsure	193242	36.5	28.3 (28.0, 28.5)	5.06 (4.94, 5.18)	3.24 (3.16, 3.32)
Missing	55629	10.5	24.9 (24.4, 25.4)	4.46 (4.33, 4.59)	2.12 (1.79, 2.45)
Continued on next page					

Extent intentionally avoiding contact with others					
All of the time	67156	12.7	11.0 (10.7, 11.3)	1.0 (NA)	1.0 (NA)
Most of the time	142287	26.9	8.4 (8.2, 8.6)	0.76 (0.73, 0.79)	0.87 (0.84, 0.90)
Some of the time	187201	35.3	9.0 (8.8, 9.1)	0.81 (0.78, 0.84)	0.88 (0.86, 0.91)
None of the time	85930	16.2	44.5 (44.1, 45.0)	4.03 (3.92, 4.15)	2.43 (2.35, 2.50)
Missing	47084	8.9	26.2 (25.6, 26.7)	2.37 (2.29, 2.45)	1.46 (1.37, 1.54)

NA=not applicable

[a] State governor's political party was excluded from the multivariable model due to collinearity with county Trump vote share.

[b] Reliable estimates could not be calculated for the missing category for variables based on participants' zip code, due to collinearity.
