# Supplementary material for: Time trends, factors associated with, and reasons for COVID-19 vaccine hesitancy: A massive online survey of US adults from January-May 2021
Source: PLoS One. 2021 Dec 21;16(12):e0260731. doi: 10.1371/journal.pone.0260731 (PMC8691631; doi:10.1371/journal.pone.0260731)
Supplement: S10 Table — (PDF) [file pone.0260731.s011.pdf]

sTable 10. Reasons for COVID-19 vaccine hesitancy in May 21 by race/ethnicity[a] among hesitant US adults (N=73362)[b]

	White	Hispanic	Black	Asian	NA	PI	Multi-racial	Unknown
N	44067	6028	3126	339	824	124	3117	15737
	% (95% CI)							
Concerned about possible side effects	51.5 (50.9, 52.1)	49.3 (47.6, 50.9)	51.4 (49.3, 53.5)	48.5 (41.6, 55.5)	41.4 (37.1, 45.7)	43.7 (33.5, 53.9)	58.5 (56.3, 60.7)	42.0 (41.0, 43.0)
Don't trust COVID-19 vaccines	50.5 (49.9, 51.0)	44.8 (43.1, 46.5)	46.1 (43.9, 48.2)	38.4 (31.5, 45.4)	45.7 (41.2, 50.1)	45.0 (34.8, 55.1)	55.1 (52.9, 57.3)	44.8 (43.7, 45.8)
Don't trust the government	43.2 (42.6, 43.8)	35.9 (34.3, 37.6)	34.3 (32.2, 36.3)	26.2 (20.1, 32.2)	42.9 (38.3, 47.5)	34.4 (24.8, 44.1)	50.0 (47.8, 52.3)	40.0 (39.0, 41.0)
Don't believe I need it	41.8 (41.2, 42.4)	32.3 (30.6, 33.9)	22.3 (20.4, 24.3)	33.1 (26.2, 40.1)	32.6 (28.6, 36.6)	28.5 (18.9, 38.1)	47.0 (44.7, 49.2)	33.8 (32.9, 34.8)
Plan to wait and to see if safe	33.9 (33.3, 34.5)	41.8 (40.1, 43.4)	41.1 (39.0, 43.2)	42.5 (35.5, 49.5)	27.0 (23.1, 30.8)	35.5 (26.0, 45.1)	34.4 (32.2, 36.5)	31.2 (30.3, 32.2)
Concerned about an allergic reaction	23.2 (22.7, 23.7)	26.6 (25.1, 28.1)	33.1 (31.0, 35.1)	26.8 (20.0, 33.6)	24.6 (21.0, 28.2)	27.4 (18.5, 36.2)	32.5 (30.4, 34.5)	21.1 (20.3, 21.9)
Don't know if it will work	22.1 (21.6, 22.6)	22.7 (21.2, 24.1)	21.2 (19.4, 23.0)	26.4 (19.4, 33.4)	17.2 (14.0, 20.3)	24.7 (16.1, 33.4)	27.6 (25.6, 29.6)	20.1 (19.2, 20.9)
Don't like vaccines	13.7 (13.3, 14.2)	15.4 (14.0, 16.8)	14.4 (12.9, 15.9)	13.7 (9.5, 17.8)	13.1 (10.5, 15.8)	14.2 (7.2, 21.3)	18.3 (16.5, 20.1)	16.1 (15.3, 16.9)
Safety concern because of my health condition	12.0 (11.7, 12.3)	11.9 (10.7, 13.0)	15.9 (14.5, 17.4)	11.8 (8.0, 15.5)	13.1 (10.8, 15.5)	10.3 (4.4, 16.2)	18.9 (17.3, 20.6)	10.6 (10.1, 11.1)
Other people need it more	11.9 (11.5, 12.3)	16.9 (15.4, 18.4)	10.4 (9.0, 11.7)	17.6 (11.6, 23.6)	10.6 (7.9, 13.2)	15.5 (8.1, 22.8)	15.7 (14.0, 17.5)	12.3 (11.6, 13.0)
Doctor has not recommended	9.0 (8.7, 9.3)	9.7 (8.5, 11.0)	7.6 (6.3, 8.9)	7.7 (4.5, 10.8)	9.5 (7.2, 11.8)	2.8 (0.2, 5.4)	14.7 (13.0, 16.3)	9.2 (8.7, 9.8)
Against religious beliefs	6.7 (6.4, 7.1)	10.3 (9.1, 11.5)	7.5 (6.4, 8.7)	5.6 (3.1, 8.1)	13.0 (10.3, 15.8)	3.1 (0.4, 5.8)	13.5 (12.0, 15.0)	9.7 (9.1, 10.3)
Currently/planning to be pregnant/breastfeeding	6.1 (5.8, 6.4)	10.0 (8.8, 11.2)	7.0 (5.9, 8.1)	11.4 (7.5, 15.3)	4.1 (2.3, 5.8)	4.1 (0.4, 7.9)	8.5 (7.2, 9.8)	6.2 (5.7, 6.6)
Concerned about cost	2.3 (2.1, 2.5)	4.9 (3.8, 6.1)	3.3 (2.5, 4.0)	3.9 (-0.2, 8.0)	2.1 (1.0, 3.2)	2.5 (-0.5, 5.4)	5.5 (4.4, 6.6)	4.3 (3.9, 4.8)
Other	15.2 (14.8, 15.6)	13.2 (12.2, 14.2)	11.5 (10.2, 12.9)	16.8 (10.6, 23.0)	19.5 (16.2, 22.7)	17.9 (10.3, 25.6)	22.9 (21.0, 24.8)	19.0 (18.3, 19.8)

NA=Native American; NH=Non-Hispanic; PI=Pacific Islander.

[a] Race/ethnicity groups other than the group labeled "Hispanic" are non-Hispanic.

[b] Answered that they probably or definitely would not choose to get vaccinated if offered a vaccine to prevent COVID-19.
